# Crosstalk between Pancreatic Cancer Cells and Cancer-Associated Fibroblasts in the Tumor Microenvironment Mediated by Exosomal MicroRNAs

**DOI:** 10.3390/ijms23179512

**Published:** 2022-08-23

**Authors:** Xiangyu Chu, Yinmo Yang, Xiaodong Tian

**Affiliations:** Department of General Surgery, Peking University First Hospital, Beijing 100034, China

**Keywords:** PDAC, exosomal miRNAs, cancer-associated fibroblasts, tumor microenvironment

## Abstract

Pancreatic ductal adenocarcinoma (PDAC) is one of the most malignant digestive tumors, characterized by a low rate of early diagnosis, strong invasiveness, and early metastasis. The abundant stromal cells, dense extracellular matrix, and lack of blood supply in PDAC limit the penetration of chemotherapeutic drugs, resulting in poor efficacy of the current treatment regimens. Cancer-associated fibroblasts (CAFs) are the major stromal cells in the tumor microenvironment. Tumor cells can secrete exosomes to promote the generation of activated CAFs, meanwhile exosomes secreted by CAFs help promote tumor progression. The aberrant expression of miRNAs in exosomes is involved in the interaction between tumor cells and CAFs, which provides the possibility for the application of exosomal miRNAs in the diagnosis and treatment of PDAC. The current article reviews the mechanism of exosomal miRNAs in the crosstalk between PDAC cells and CAFs in the tumor microenvironment, in order to improve the understanding of TME regulation and provide evidence for designing diagnostic and therapeutic targets against exosome miRNA in human PDAC.

## 1. Introduction

The incidence and associated mortality of pancreatic ductal adenocarcinoma (PDAC) are still steadily increasing, which is mainly due to its extremely poor prognosis. Cancer-related mortality of this dismal disease ranks seventh globally and is sixth in China [1,2]. Because of the insidious onset and the unspecific clinical manifestations, it is difficult to diagnose PDAC at an early stage; hence, only 20% of patients are amenable to surgical treatment [3]. Early diagnosis is essential to increasing the resection rate and improving the overall prognosis of PDAC, which requires more sensitive serum diagnostic markers. However, the sensitivity and specificity of the current biomarkers, including CEA, CA19-9, CA24-2, and CA125, cannot meet clinical demands in the diagnosis of PDAC; hence, new biomarkers need to be developed. Another important feature affecting the prognosis of PDAC is the prominent desmoplastic change, which contributes its insensitivity to current radiochemotherapy. One of the recent research hotspots is discovering the complex composition of the tumor microenvironment (TME) and developing novel targets to improve the therapeutic effect of PDAC. Tumor stroma of PDAC is characterized by tissue fibrosis, hypoxia, and an immunosuppressive microenvironment, of which activated cancer-associated fibroblasts (CAFs) represent the major cellular component [4]. Intercommunication between cancer cells and CAFs facilitates PDAC progression, metastasis, and chemoresistance. In recent years, the role of exosomes in the intercellular crosstalk has attracted increasing attention.

**Figure 1:**
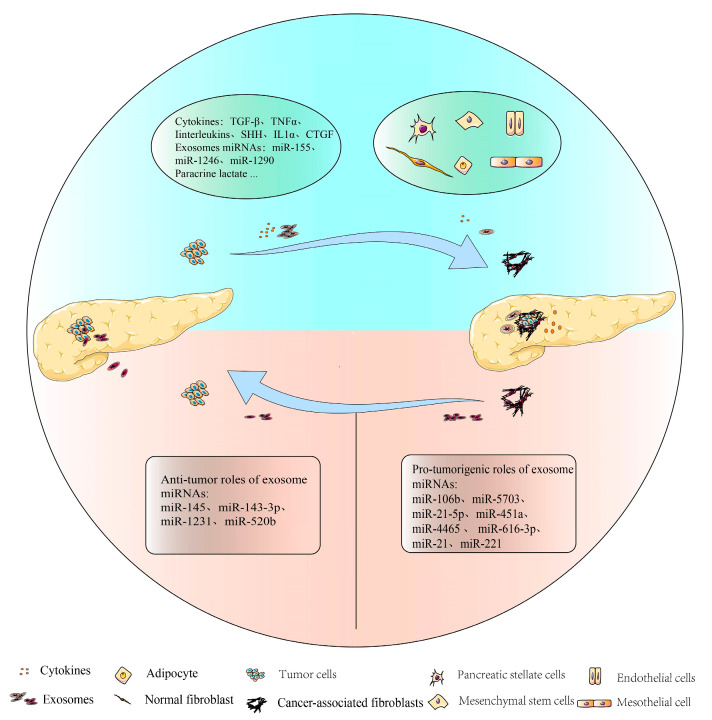
Exosomal miRNAs are involved in the crosstalk between PDAC cells and cancer-associated fibroblasts.

Exosomes are small, lipid bilayer membrane vesicles of endocytic origin with a diameter of 30–150 nm, which are widely present in blood, ascites, urine, and saliva [5,6]. Exosomes contain biomolecules derived from parental cells, including DNA, microRNAs (miRNAs), long non-coding RNAs (lncRNAs), proteins, and other metabolites, which play an important role in intercellular communication and the regulation of physiological and pathological processes [7,8,9,10,11]. MiRNAs are a class of non-coding RNAs consisting of 19–24 nucleotides that inhibit the expression of downstream target genes by interfering with mRNA translation or promoting its degradation. In the TME, miRNAs can be delivered to cancer and stromal cells via exosomes to promote intercellular communication and facilitate cancer progression such as proliferation, invasion, and metastasis. Aberrant expression of miRNAs in exosomes is not only involved in the regulation of cellular communication between tumor cells and fibroblasts, but also in the formation of the immunosuppressive microenvironment in PDAC. The complexity of their functions provides the possibility for the application in the diagnosis of PDAC. Specific molecules including miRNA, lncRNA, and proteins are enriched and preserved in various parental cell-derived exosomes which could serve as potential biomarkers for cancer diagnosis. Moreover, accumulating evidence proves that exosomal miRNAs are involved in the interaction between tumor and stromal cells in the TME of PDAC, indicating its potential to improve in the diagnosis and treatment of PDAC. In this article, we review the current knowledge on exosomal miRNAs involving the intercellular communication between tumor and stromal cells, in order to improve the understanding of TME regulation and provide evidence for designing diagnostic and therapeutic targets against exosome miRNA in human PDAC (Figure 1).

## 2. Roles of CAFs in the TME of PDAC

### 2.1. Identification, Origin, and Classification of CAFs in PDAC

In PDAC, 60–70% of the tumor tissue is composed of CAF-dominant stromal cells and the deposition of extracellular matrix (ECM) components such as collagen [12]. CAFs are characterized by the expression of mesenchymal markers, morphological features (spindle shape), and by the absence of non-mesenchymal markers (markers of epithelial, endothelial, immune, and neuronal cells). The latest consensus states that CAFs are collections of cells in the tumors that are negative for epithelial, endothelial, and leukocyte markers; have an elongated morphology; and lack the genetic mutations found in cancer cells [13]. CAFs can be derived from normal fibroblasts, stellate cells, bone marrow-derived mesenchymal stem cells, adipocytes, or endothelial cells [14,15,16,17] (Figure 1). Quiescent PSCs are the main precursors of CAFs in the TME of PDAC. When injury or inflammation occurs, quiescent PSCs will be activated, accompanied by changes in cell morphology and the disappearance of lipid droplets. Subsequently, quiescent PSCs transform into α-smooth muscle actin (α-SMA)-positive myofibroblast-like cells, secreting a large amount of extracellular matrix components and remodeling the matrix of pancreatic tissue [18]. Matthew et al. [19] reported that the fibroblast subpopulations exist in both normal pancreas and pancreatic tumors in KC (Ptf1a-Cre; LSL-Kras G12D) mice, and these fibroblast subpopulations display characteristics of mesenchymal stem cells (MSCs), which can differentiate into chondrocytes, adipocytes, and osteoblasts. This would promote tumor growth by inducing the polarization of macrophages to pro-cancer subtypes. Huang et al. [20] found mesothelial cells could transform into antigen-presenting CAFs (apCAFs) by downregulating mesothelial characteristics and acquiring fibroblast characteristics under the induction of interleukin-1 and transforming growth factor β (TGF-β). The apCAFs can directly ligate and convert the naive CD4+ T cells into regulatory T cells in an antigen-specific way. Miyazaki et al. [21] found that the adipose-derived mesenchymal stem cells (AD-MSCs) could transform into variant CAF subtypes under different co-culture conditions in vitro. When co-cultured with PDAC Capan-1 cells directly, AD-MSCs could transform into myofibroblast CAFs (myCAFs) and inflammatory CAFs (iCAFs), while indirect co-culture induced differentiation into only iCAFs. Waghray et al. [22] identified and characterized cancer-associated MSC (CA-MSC) subpopulations from human PDAC samples and CAFs, highlighting the heterogeneity of fibroblast subpopulations in PDAC. CA-MSCs can significantly enhance the proliferation, invasion, and metastatic potential of PDAC cells by secreting granulocyte-macrophage colony-stimulating factor (GM-CSF). CAFs will then differentiate into different subtypes with similar characteristics to their origin cells, according to the various cell origins and culture conditions. Hence, CAFs in human PDAC show great heterogeneity and exert different functions.

### 2.2. Roles of Activated CAFs in the PDAC Microenvironment

The activated CAFs can express various proteins including α-SMA, fibroblast activation protein (FAP), fibroblast-specific protein 1 (FSP-1), podoplanin (PDPN), and PDGFR [16,23]. Some of these proteins were used as typing markers to classify CAFs into different subtypes, which might exert anti-cancer or pro-cancer roles in PDAC. CAFs with high expression of α-SMA were named myCAFs, whereas CAFs with low α-SMA levels that secrete inflammatory cytokines such as IL-6 and leukemia inhibitory factor (LIF) were defined as iCAF [24]. McAndrews et al. [25] found that depletion of FAP+ CAFs resulted in a survival benefit, while depletion of α-SMA+ CAFs caused increased mortality in mouse models of PDAC. The oncogenic FAP+ CAFs and anti-tumor α-SMA+ CAFs were proved to regulate cancer-related pathways and regulatory T cells via different mechanisms. Elyada et al. [26] identified a subtype of apCAFs with MHCII expression and antigen-presenting capability in KPC PDAC mice, which could present the model antigen to the CD4+ T cells, decrease the CD8+T/Treg ratio, and contribute to immune suppression in the TME. The apCAFs could also transform into myCAFs under appropriate culture conditions, which implied that CAFs were in a dynamic cell state. Lin et al. [27] confirmed the presence of three CAF subtypes (myCAF, iCAF, and apCAF) in human PDAC tissues by single-cell transcriptomic technology. The subtype of CAFs with high Meflin expression was reported to correlate with a favorable outcome in both mouse models or patients with PDAC, and deficiency or low expression of Meflin resulted in straightened stromal collagen fibers, indicating that Meflin was a marker of cancer-restraining CAFs that suppressed the progression of PDAC [28]. Chen et al. [29] identified a subset of complement-secreting CAFs (csCAFs) which were located in the tissue stroma adjacent to tumor cells only in early PDAC and specifically expressed complement system components.

PSCs were found to produce myeloid-derived suppressor cell (MDSC)-promoting cytokines (interleukins, IL-6; VEGF; macrophage colony-stimulating factor, M-CSF) and chemokines (SDF-1, MCP-1) to promote differentiation of immune cells into the functional MDSC phenotypes to prevent innate or adaptive immune responses against cancer cells, among which IL-6 was the key factor contributing to STAT3 signaling and MDSC differentiation [30]. CAFs were also reported to upregulate the expression of immune checkpoints PD-1, cytotoxic lymphocyte-associated antigen-4 (CTLA-4), T-cell immunoglobulin, mucin-domain containing-3 (TIM-3), and lymphocyte-activation gene-3 (LAG-3) in both CD4+ and CD8+ T cells, thereby inhibiting the proliferation of T cells and diminishing immune function in PDAC [31]. 

The specific mechanisms by which CAFs regulate tumor progression have not yet been fully clarified. More and more evidence has shown that the activation mechanisms of different CAF subtypes varied widely, and the dominant subtype of CAFs could dynamically change in response to different biological signals in the TME of PDAC or different in vitro culture conditions [21,32]. Along with the understanding of the differentiation and regulational mechanism of CAFs and their effect on tumor cells in the TME of PDAC, novel therapeutic drugs targeting CAFs modulation may be developed to transform the tumor-promoting microenvironment into a tumor-suppressing microenvironment, thereby increasing the sensitivity of immunotherapy.

## 3. Regulatory Mechanism of CAFs Differentiation and Activation in PDAC

In a healthy pancreas, fibroblasts exist mainly in a quiescent state. During the progress of carcinogenesis, quiescent fibroblasts transform into active CAFs through the activation of distinct signaling pathways with the participation of miRNAs, cytokines, paracrine lactate, and growth factors secreted by tumor cells (Figure 1). 

### 3.1. Tumor Cells Promote the Differentiation and Activation of CAFs

Tumor cells can secrete connective tissue growth factor (CTGF) to promote the activation, proliferation, migration, and adhesion of PSCs and fibroproliferation [33]. Biffi et al. [34] found that PDAC cells could secrete IL1α to activate PSCs and promote the formation of iCAFs by activating the IL1/JAK/STAT signaling pathway, and tumor cells could also increase the expression of inflammatory cytokines and chemokines like IL1a, IL6, and LIF in iCAFs, which promoted cancer progression and formed an immunosuppressive microenvironment. Moreover, PDAC cells secreted TGFβ to upregulate the expression of α-SMA in PSCs, and thus transformed the quiescent PSCs to myCAF, promoting the formation of a dense extracellular matrix [34]. The presence of oncogenic KRAS mutation was also reported to be involved in the activation of CAFs and to regulate tumor cell signaling via stromal cells [35]. Awaji [36] found that KRAS mutation could promote the secretion of paracrine factors such as IL4, IL10, and IL13 in tumor cells and simultaneously activate CXCR2 signaling in CAFs, resulting in the formation of a secreted CAF phenotype by activating NF-κB signaling and promoting tumor progression. The paracrine lactate secreted by PDAC cells could increase alpha-ketoglutarate (aKG) production in MSCs, which decreased cytosine methylation and increased hydroxymethylation during the differentiation of MSCs into CAFs by mediating the activation of the demethylase enzyme ten-eleven translocation (TET) [37]. Additionally, the reactive oxygen species (ROS) associated with the hypoxic TME of PDAC could also promote the activation of PSCs by stabilizing HIF-1α and increasing the expression of GLI1 [38].

### 3.2. Aberrant Expression of miRNAs Was Involved in the Activation of CAFs

Studies have revealed that the aberrant expression of miRNAs in PDAC played important roles in the activation of CAFs in the TME. Xu et al. [39] reported that miR-200a could attenuate the activation of PSC induced by TGF-β1 and inhibit ECM formation through the PTEN/Akt/mTOR pathway. Inhibition of miR-199a-3p and miR-214-3p was found to be involved in the de-differentiation of CAFs which could reduce the differentiation of PSCs into myofibroblasts [40]. Chu et al. indicated that the overexpression of miR-224 in pancreatic fibroblasts can significantly increase their proliferation, migration, and invasion ability, thus promoting the activation of CAFs [41]. Overexpression of miR-21 contributed to the activation of CAFs by regulating the PDCD4 gene and increased the invasion ability of PDAC cells by stimulating the secretion of MMP-3, MMP-9, CCL-7, and PDGF in CAFs [42]. In a mouse model of alcoholic chronic pancreatitis (CP), Charrier et al. [43] reported that the expression of miR-21 was significantly elevated in activated PSCs, which promoted the expression of connective tissue growth factor (CCN2), and a positive feedback loop between miR-21 and CCN2 was found to increase the expression of collagen and the activation of quiescent PSCs. MiR-301a was also involved in the activation of PSCs by inhibiting Gadd45g expression and promoting STAT3 activation during the pancreatic intraepithelial neoplasia lesion formation [44]. 

### 3.3. Exosomal miRNAs Promote the Activation of CAFs in TME of PDAC 

The specifically enriched miRNAs in the exosomes play vital roles as messengers of cell-to-cell interaction in the TME (Figure 1). Pang et al. [45] reported that PDAC cells delivered miR-155 into fibroblasts via exosomes, which could inhibit the expression of the target gene TP53INP1 and reprogram the quiescent fibroblasts into CAFs. MiR-1246 and miR-1290 were highly enriched in PDAC cell-derived exosomes, which could be transferred into PSCs to induce the activation of PSCs and the occurrence of pro-fibrosis, including proliferation, migration, collagen production, and expression of α-SMA [46].

In summary, the activation of CAFs is essential to form the immunosuppressive TME in PDAC. In the process of carcinogenesis, the differentiation and activation of CAFs was modulated by complex mechanisms, among which exosomal miRNAs derived from tumor cells play important roles, although the specific mechanism remains to be further studied.

## 4. Exosomal miRNAs Derived from CAFs Regulate the Malignant Phenotype of PDAC Cells

Different subtypes of CAFs are functionally heterogeneous and play different roles in cancer development [28]. CAF-derived exosomes have been reported to be involved in regulating the malignant behavior of PDAC cells, among which the abnormally expressed miRNAs secreted by CAFs play the important role of either pro-tumor or anti-tumor executors by targeting downstream genes (Figure 1).

### 4.1. CAF-Derived Exosomal miRNAs Could Promote PDAC Progression

In 2017, Richards and colleagues [7] found that gemcitabine could significantly stimulate CAFs to secrete more exosomes, of which miR-146a was highly enriched, thus promoting PDAC proliferation and gemcitabine resistance. Likewise, Fang et al. [47] also reported that CAF-derived exosomal miR-106b could increase the resistance of PDAC cells to gemcitabine by targeting TP53INP1. CAF-derived exosomal miRNAs could also promote the proliferation and metastasis of PDAC. Li et al. [8] found that PSC-derived exosomal miR-5703 could target CKLF-like MARVEL transmembrane domain 4 (CMTM4) to inhibit its expression in PDAC cells, thus upregulating p21-activated kinase 4 (PAK4) expression and activating the PI3K/Akt pathway, promoting cancer cell proliferation. PSC-derived exosomes were also found to specifically enrich miR-21-5p and miR-451a, to promote the proliferation, invasion, and metastasis of PDAC cells [48]. Cao et al. [49] reported that a hypoxic TME could upregulate the expression of miR-4465 and miR-616-3p in PSC exosomes, which promoted PDAC progression and metastasis by inhibiting PTEN and activating the AKT pathway. Ali and colleagues [50] found that both PSC-derived exosomal miR-21 and CAF-derived exosomal miR-221 could promote the proliferation, migration, and invasion of PDAC cells, and miR-221 was associated with promoting KRAS and NF-κB. The active CAFs in TME secrete exosomal miRNAs to promote PDAC progression, meanwhile tumor cells constantly retain the activation of CAFs, thus forming a feedback loop that is favorable for tumor progression.

### 4.2. CAFs Secrete Exosomal miRNAs to Inhibit PDAC Progression

In addition to the pro-oncogenic role of CAFs in the TME, some studies also confirmed the anti-tumor effect of CAFs in PDAC, and exosomal miRNAs were the key mediators of intercellular crosstalk. Stroma-derived extracellular vesicles could deliver tumor-suppressive miR-145 to induce apoptosis and inhibit the proliferation of PDAC cells [51]. Human MSC-derived exosomal miR-143-3p was reported to promote PDAC apoptosis and inhibit proliferation, invasion, and migration of PDAC both in vitro and in vivo [52]. In addition, overexpression of circRNA-0030167 in MSC exosomes was reported to inhibit miR-338-5p expression, promote the expression of WNT inhibitory factor 1 (WIF1), and inhibit the WNT8/β-catenin pathway, thereby inhibiting the stemness and tumor progression of PDAC [53]. Exosomal miR-1231 from bone marrow-derived mesenchymal stem cells was also found to inhibit the proliferation, migration, invasion, and adhesion of PDAC cells [54].

The CAFs in the TME of PDAC play paradoxical roles, indicating the cell competition feature and negative feedback loop to maintain the relatively stable proliferation state of the tumor. Targeting this internal mechanism of TME to amplify the tumor-suppressing effect of CAFs may provide a promising novel anti-tumor strategy. However, further studies are necessary to identify the exact function of each CAF subtype and to fully understand the mechanism of intercellular crosstalk inside the TME of PDAC.

## 5. The Current Application of TME-Related Exosomal miRNAs in the Diagnosis and Treatment of PDAC

The aberrant expression of exosomal miRNAs could not only regulate the interaction between tumor cells and CAFs, but also participate in the formation of the immunosuppressive microenvironment of PDAC. Moreover, exosomal miRNAs are also involved in the progression of PDAC-related inflammatory or metabolic disorders, like CP and diabetes mellitus [55,56]. Despite their functional complexity, some studies have demonstrated the possibility of the application of TME-related exosomal miRNAs in the diagnosis and treatment of PDAC.

### 5.1. The Diagnostic Application of TME-Related Exosomal miRNAs in PDAC

MiRNAs are specifically enriched in exosomes due to the strict sorting mechanism and protective effects of exosomes for miRNAs from degradation by RNases. Therefore, semiquantitative and quantitative detection of specific exosomal miRNAs from blood or other body fluids is considered as a promising method of liquid biopsy. The TME-related exosomal miRNAs from peripheral blood, portal blood, saliva, or pancreatic juice that have been reported to serve as diagnostic biomarkers of PDAC are summarized in Table 1. In 2016, Machida et al. [57] reported that miR1246 and miR4644 in salivary exosome were potential biomarkers for pancreatobiliary tract cancer, which could differentiate pancreaticobiliary tract cancer patients (*n* = 12) from healthy controls (*n* = 13), with a diagnostic sensitivity of 83.3% and specificity of 92.3%, and the area under the curve (AUC) = 0.833. Likewise, plasma exosome miR-196a and miR-1246 were reported as potential indicators for localized PDAC [58]. Lai et al. [59] found that a miRNA signature with high levels of exosomal miR-10b, miR-21, miR-30c, and miR-181a and low exosomal miR-let7a was superior to exosomal glypican-1 or plasma CA 19-9 for diagnosing PDAC and differentiating between PDAC and CP. In addition, an elevated expression of serum exosomal miR-191, miR-21, and miR-451a of PDAC was also considered to be an efficient diagnostic marker [60]. Wu et al. [61] reported that the combination of CA19-9 with exosomal miRNA-21 or exosomal miRNA-210 could significantly improve the diagnostic sensitivity and specificity, indicating that combination of multiple exosomal miRNAs or exosomal miRNAs with proteins could further improve the diagnostic efficiency. Recently, Li et al. [8] reported that exosomal miR-5703 in the peripheral blood of PDAC patients (*n* = 23) was significantly higher than that in healthy controls (*n* = 17). Serum exosomal miR-451a could also effectively differentiate PDAC (*n* = 8) from healthy controls (n = 8), with a dignostic sensitivity of 80.10%, specificity of 86.67%, and AUC = 0.896 [62]. In 2019, Kawamura et al. [63] reported that exosomal miR-4525, miR-451a, and miR-21 in portal vein blood was a significantly more sensitive liquid biomarker for the diagnosis and differentiation of PDAC, indicating that exosomal miRNAs in portal vein blood were more valuable than peripheral blood. Nakamura et al. [64] detected exosomal miR-21 and miR-155 in the pancreatic juice samples of 27 PDAC and 8 CP patients, and found that the levels of both exosomal miR-21 and miR-155 (but not the levels in whole pancreatic juice) were significantly higher in PDAC patients compared with CP patients and could discriminate PDAC patients from CP patients with AUC values of 0.90 and 0.89, respectively. Moreover, the combination of exosomal miR profiling with pancreatic juice cytology could improve the diagnostic accuracy of PDAC to 91% [64]. On the other hand, Que et al. [65] found that the expression of exosomal miR-155 in the serum of PDAC patients was significantly decreased, suggesting that exosomal miRNAs might be distributed differently in variant body fluids.

It should be noted that there has been no research specifically targeting TME-related exosomal miRNAs as diagnostic tools of pancreatic cancer, and the exact cellular origin of these exosomal miRNAs was unclear. Moreover, some of these exosomal miRNAs (for example, miR21 [69]) were also correlated with inflammatory or metabolic diseases, resulting in the possibility of inaccuracy for the diagnosis of pancreatic cancer. All these issues indicate that the diagnostic application of TME-related exosomal miRNAs in human PDAC is still far away.

### 5.2. The Application Prospect of Exosome-Delivered miRNAs in Treatment of PDAC

MiRNAs have important roles in the regulation of gene expression and TME. Nucleic acid drugs include antisense oligonucleotides, small interfering RNAs, miRNAs, aptamers, decoys, and CpG oligodeoxynucleotides, which might be used to achieve precise therapy for human cancers. Xie et al. [70] constructed a local delivery system for the combination of triple miR-210/siKRASG12D nanotherapy based on cholesterol-modified polymeric CXCR4 antagonist, which could specifically kill tumor cells (induced by siKRASG12D), inactivate PSCs (induced by miR-210), and block the cancer-stroma interaction (induced by CXCR4 antagonist), resulting in modulating the dense tumor microenvironment, promoting the infiltration of cytotoxic T cells, and inhibiting PDAC progression. Gilles et al. [71] designed the targeted nanoparticles iRGD-TPN-21, which delivered anti-miR-21 to inhibit the progression of PDAC. These miRNAs-based nucleic acid drugs have been proved to be promising for PDAC treatment. However, due to the widespread presence of nucleases, nanoparticles are easily degraded and have poor targeting properties, resulting in low bioavailability.

The exosome membrane can effectively protect miRNAs from degradation by nucleases. Exosomes from human umbilical cord MSC could deliver exogenous miR-145-5p to specifically downregulate the expression of Smad3, thereby inhibiting the proliferation and invasion, and promoting apoptosis of PDAC cells [72]. Zuo et al. [73] encapsulated the miR-34a into HEK293 cell-derived exosomes by sonication, and found that the synthesized exosomal miR-34a can be taken up by tumor cells and downregulate the expression of the target gene Bcl-2 in PDAC cells, both in vitro and in vivo. In 2016, Su et al. [74] used synthetic hyaluronic acid-poly (ethylene imine) and hyaluronic acid-poly (ethylene glycol) self-assembled nanoparticles to encapsulate and deliver plasmid DNA expressing miR-155 and miR-125b into Panc-1 cells, and these miR-155 and miR-125b enriched exosomes could promote macrophage reprogramming from M2 phenotype to M1 phenotype in the TME of PDAC [75]. The application of exosome-delivered miRNAs in the treatment of PDAC has shown promising prospects, but there are still great challenges in the clinical translation.

## 6. Conclusions and Prospects

TME is a highly complex, heterocellular ecosystem, consisting of tumor cells and the surrounding blood vessels, immunocytes, CAFs, and extracellular matrix. Exosomes are essential to conduct substance exchange, energy flow, and signal transmission between different cell components in the TME. The differentiation and activation of CAFs is an important event in the development of PDAC, and this process is regulated by tumor cells, partly through exosomal miRNAs. In turn, the CAFs in the TME also influence the tumor cells via either pro- or anti-tumor effects, resulting in a relatively stable state that promotes tumor progression. The regulational mechanism of this crosstalk process, of which exosomal miRNAs are only a small part, remains far from fully understood. Currently, there are few studies focusing on the mechanism of exosomal miRNAs in the regulation of CAF differentiation, and the specific exosomal miRNAs secreted by particular subtypes of CAFs to promote or suppress tumor progression still need to be further studied. In conclusion, the investigation of exosomal miRNAs that are involved in the interaction between tumor cells and different CAF subtypes and their regulatory mechanisms will have broad application prospects, not only in tumor liquid biopsy, but also in novel targeted therapy strategies for PDAC.

## Figures and Tables

**Table 1:** The aberrant expression and distribution of TME-related exosomal miRNAs in PDAC.

Exosomal miRNA	Expression	Portal Vein	Plasma or Serum	Pancreatic Juice	Saliva
miR-1246	Upregulation		[57,58]		[57]
miR-5703	Upregulation		[8]		
miR-451a	Upregulation	[63]	[60,62]		
miR-21	Upregulation	[63]	[59,61]	[65,66]	
miR-221	Upregulation		[67]		
miR-1231	Downregulation		[68]		
miR-155	Upregulation Downregulation		[65]	[64]	

## Data Availability

Not applicable.

## References

[1] Bray F., Ferlay J., Soerjomataram I., Siegel R.L., Torre L.A., Jemal A. (2018). Global cancer statistics 2018: GLOBOCAN estimates of incidence and mortality worldwide for 36 cancers in 185 countries. CA Cancer J. Clin..

[2] Ferlay J., Ervik M., Lam F., Colombet M., Mery L., Piñeros M., Znaor A., Soerjomataram I., Bray F. (2020). Global Cancer Observatory: Cancer Today.

[3] Khorana A.A., Mangu P.B., Berlin J., Engebretson A., Hong T.S., Maitra A., Mohile S.G., Mumber M., Schulick R., Shapiro M. (2016). Potentially Curable Pancreatic Cancer: American Society of Clinical Oncology Clinical Practice Guideline. J. Clin. Oncol..

[4] Cortesi M., Zanoni M., Pirini F., Tumedei M.M., Ravaioli S., Rapposelli I.G., Frassineti G.L., Bravaccini S. (2021). Pancreatic Cancer and Cellular Senescence: Tumor Microenvironment under the Spotlight. Int. J. Mol. Sci..

[5] Kalluri R., LeBleu V.S. (2020). The biology, function, and biomedical applications of exosomes. Science.

[6] Kalluri R. (2016). The biology and function of exosomes in cancer. J. Clin. Investg..

[7] Richards K.E., Zeleniak A.E., Fishel M.L., Wu J., Littlepage L.E., Hill R. (2017). Cancer-associated fibroblast exosomes regulate survival and proliferation of pancreatic cancer cells. Oncogene.

[8] Li M., Guo H., Wang Q., Chen K., Marko K., Tian X., Yang Y. (2020). Pancreatic stellate cells derived exosomal miR-5703 promotes pancreatic cancer by downregulating CMTM4 and activating PI3K/Akt pathway. Cancer Lett..

[9] Yang B., Feng X., Liu H., Tong R., Wu J., Li C., Yu H., Chen Y., Cheng Q., Chen J. (2020). High-metastatic cancer cells derived exosomal miR92a-3p promotes epithelial-mesenchymal transition and metastasis of low-metastatic cancer cells by regulating PTEN/Akt pathway in hepatocellular carcinoma. Oncogene.

[10] Xie M., Yu T., Jing X., Ma L., Fan Y., Yang F., Ma P., Jiang H., Wu X., Shu Y. (2020). Exosomal circSHKBP1 promotes gastric cancer progression via regulating the miR-582-3p/HUR/VEGF axis and suppressing HSP90 degradation. Mol. Cancer.

[11] Wang L., Bo X., Yi X., Xiao X., Zheng Q., Ma L., Li B. (2020). Exosome-transferred LINC01559 promotes the progression of gastric cancer via PI3K/AKT signaling pathway. Cell Death Dis..

[12] Ziani L., Chouaib S., Thiery J. (2018). Alteration of the Antitumor Immune Response by Cancer-Associated Fibroblasts. Front. Immunol..

[13] Sahai E., Astsaturov I., Cukierman E., DeNardo D.G., Egeblad M., Evans R.M., Fearon D., Greten F.R., Hingorani S.R., Hunter T. (2020). A framework for advancing our understanding of cancer-associated fibroblasts. Nat. Rev. Cancer.

[14] Kanzaki R., Pietras K. (2020). Heterogeneity of cancer-associated fibroblasts: Opportunities for precision medicine. Cancer Sci..

[15] Bu L., Baba H., Yoshida N., Miyake K., Yasuda T., Uchihara T., Tan P., Ishimoto T. (2019). Biological heterogeneity and versatility of cancer-associated fibroblasts in the tumor microenvironment. Oncogene.

[16] Kobayashi H., Enomoto A., Woods S.L., Burt A.D., Takahashi M., Worthley D.L. (2019). Cancer-associated fibroblasts in gastrointestinal cancer. Nat. Rev. Gastroenterol. Hepatol..

[17] Nurmik M., Ullmann P., Rodriguez F., Haan S., Letellier E. (2020). In search of definitions: Cancer-associated fibroblasts and their markers. Int. J. Cancer.

[18] Bynigeri R.R., Jakkampudi A., Jangala R., Subramanyam C., Sasikala M., Rao G.V., Reddy D.N., Talukdar R. (2017). Pancreatic stellate cell: Pandora’s box for pancreatic disease biology. World J. Gastroenterol..

[19] Mathew E., Brannon A.L., Del Vecchio A., Garcia P.E., Penny M.K., Kane K.T., Vinta A., Buckanovich R.J., di Magliano M.P. (2016). Mesenchymal Stem Cells Promote Pancreatic Tumor Growth by Inducing Alternative Polarization of Macrophages. Neoplasia.

[20] Huang H., Wang Z., Zhang Y., Pradhan R.N., Ganguly D., Chandra R., Murimwa G., Wright S., Gu X., Maddipati R. (2022). Mesothelial cell-derived antigen-presenting cancer-associated fibroblasts induce expansion of regulatory T cells in pancreatic cancer. Cancer Cell.

[21] Miyazaki Y., Oda T., Mori N., Kida Y.S. (2020). Adipose-derived mesenchymal stem cells differentiate into pancreatic cancer-associated fibroblasts in vitro. FEBS Open Bio.

[22] Waghray M., Yalamanchili M., Dziubinski M., Zeinali M., Erkkinen M., Yang H., Schradle K.A., Urs S., Pasca Di Magliano M., Welling T.H. (2016). GM-CSF Mediates Mesenchymal-Epithelial Cross-talk in Pancreatic Cancer. Cancer Discov..

[23] Park D., Sahai E., Rullan A. (2020). SnapShot: Cancer-Associated Fibroblasts. Cell.

[24] Ohlund D., Handly-Santana A., Biffi G., Elyada E., Almeida A.S., Ponz-Sarvise M., Corbo V., Oni T.E., Hearn S.A., Lee E.J. (2017). Distinct populations of inflammatory fibroblasts and myofibroblasts in pancreatic cancer. J. Exp. Med..

[25] McAndrews K.M., Chen Y., Darpolor J.K., Zheng X., Yang S., Carstens J.L., Li B., Wang H., Miyake T., Correa de Sampaio P. (2022). Identification of Functional Heterogeneity of Carcinoma-Associated Fibroblasts with Distinct IL6-Mediated Therapy Resistance in Pancreatic Cancer. Cancer Discov..

[26] Elyada E., Bolisetty M., Laise P., Flynn W.F., Courtois E.T., Burkhart R.A., Teinor J.A., Belleau P., Biffi G., Lucito M.S. (2019). Cross-Species Single-Cell Analysis of Pancreatic Ductal Adenocarcinoma Reveals Antigen-Presenting Cancer-Associated Fibroblasts. Cancer Discov..

[27] Lin W., Noel P., Borazanci E.H., Lee J., Amini A., Han I.W., Heo J.S., Jameson G.S., Fraser C., Steinbach M. (2020). Single-cell transcriptome analysis of tumor and stromal compartments of pancreatic ductal adenocarcinoma primary tumors and metastatic lesions. Genome Med..

[28] Mizutani Y., Kobayashi H., Iida T., Asai N., Masamune A., Hara A., Esaki N., Ushida K., Mii S., Shiraki Y. (2019). Meflin-Positive Cancer-Associated Fibroblasts Inhibit Pancreatic Carcinogenesis. Cancer Res..

[29] Chen K., Wang Q., Li M., Guo H., Liu W., Wang F., Tian X., Yang Y. (2021). Single-cell RNA-seq reveals dynamic change in tumor microenvironment during pancreatic ductal adenocarcinoma malignant progression. EBioMedicine.

[30] Mace T.A., Ameen Z., Collins A., Wojcik S., Mair M., Young G.S., Fuchs J.R., Eubank T.D., Frankel W.L., Bekaii-Saab T. (2013). Pancreatic cancer-associated stellate cells promote differentiation of myeloid-derived suppressor cells in a STAT3-dependent manner. Cancer Res..

[31] Gorchs L., Fernandez Moro C., Bankhead P., Kern K.P., Sadeak I., Meng Q., Rangelova E., Kaipe H. (2019). Human Pancreatic Carcinoma-Associated Fibroblasts Promote Expression of Co-inhibitory Markers on CD4(+) and CD8(+) T-Cells. Front. Immunol..

[32] Sunami Y., Haussler J., Kleeff J. (2020). Cellular Heterogeneity of Pancreatic Stellate Cells, Mesenchymal Stem Cells, and Cancer-Associated Fibroblasts in Pancreatic Cancer. Cancers.

[33] Charrier A., Brigstock D.R. (2013). Regulation of pancreatic function by connective tissue growth factor (CTGF, CCN2). Cytokine Growth Factor Rev..

[34] Biffi G., Oni T.E., Spielman B., Hao Y., Elyada E., Park Y., Preall J., Tuveson D.A. (2019). IL1-Induced JAK/STAT Signaling Is Antagonized by TGFbeta to Shape CAF Heterogeneity in Pancreatic Ductal Adenocarcinoma. Cancer Discov..

[35] Tape C.J., Ling S., Dimitriadi M., McMahon K.M., Worboys J.D., Leong H.S., Norrie I.C., Miller C.J., Poulogiannis G., Lauffenburger D.A. (2016). Oncogenic KRAS Regulates Tumor Cell Signaling via Stromal Reciprocation. Cell.

[36] Awaji M., Saxena S., Wu L., Prajapati D.R., Purohit A., Varney M.L., Kumar S., Rachagani S., Ly Q.P., Jain M. (2020). CXCR2 signaling promotes secretory cancer-associated fibroblasts in pancreatic ductal adenocarcinoma. FASEB J..

[37] Bhagat T.D., Von Ahrens D., Dawlaty M., Zou Y., Baddour J., Achreja A., Zhao H., Yang L., Patel B., Kwak C. (2019). Lactate-mediated epigenetic reprogramming regulates formation of human pancreatic cancer-associated fibroblasts. Elife.

[38] Lei J., Huo X., Duan W., Xu Q., Li R., Ma J., Li X., Han L., Li W., Sun H. (2014). alpha-Mangostin inhibits hypoxia-driven ROS-induced PSC activation and pancreatic cancer cell invasion. Cancer Lett..

[39] Xu M., Wang G., Zhou H., Cai J., Li P., Zhou M., Lu Y., Jiang X., Huang H., Zhang Y. (2017). TGF-beta1-miR-200a-PTEN induces epithelial-mesenchymal transition and fibrosis of pancreatic stellate cells. Mol. Cell Biochem..

[40] Kuninty P.R., Bojmar L., Tjomsland V., Larsson M., Storm G., Ostman A., Sandstrom P., Prakash J. (2016). MicroRNA-199a and -214 as potential therapeutic targets in pancreatic stellate cells in pancreatic tumor. Oncotarget.

[41] Chu N.J., Anders R.A., Fertig E.J., Cao M., Hopkins A.C., Keenan B.P., Popovic A., Armstrong T.D., Jaffee E.M., Zimmerman J.W. (2020). Inhibition of miR-21 Regulates Mutant KRAS Effector Pathways and Intercepts Pancreatic Ductal Adenocarcinoma Development. Cancer Prev. Res..

[42] Zhang L., Yao J., Li W., Zhang C. (2018). Micro-RNA-21 Regulates Cancer-Associated Fibroblast-Mediated Drug Resistance in Pancreatic Cancer. Oncol. Res..

[43] Charrier A., Chen R., Chen L., Kemper S., Hattori T., Takigawa M., Brigstock D.R. (2014). Connective tissue growth factor (CCN2) and microRNA-21 are components of a positive feedback loop in pancreatic stellate cells (PSC) during and are exported in PSC-derived exosomes. J. Cell Commun. Signal..

[44] Li F., Wang M., Li X., Long Y., Chen K., Wang X., Zhong M., Cheng W., Tian X., Wang P. (2022). Inflammatory-miR-301a circuitry drives mTOR and Stat3-dcotarget PSC activation in chronic pancreatitis and PanIN. Mol. Ther.-Nucleic Acids.

[45] Pang W., Su J., Wang Y., Feng H., Dai X., Yuan Y., Chen X., Yao W. (2015). Pancreatic cancer-secreted miR-155 implicates in the conversion from normal fibroblasts to cancer-associated fibroblasts. Cancer Sci..

[46] Masamune A., Yoshida N., Hamada S., Takikawa T., Nabeshima T., Shimosegawa T. (2018). Exosomes derived from pancreatic cancer cells induce activation and profibrogenic activities in pancreatic stellate cells. Biochem. Biophys. Res. Commun..

[47] Fang Y., Zhou W., Rong Y., Kuang T., Xu X., Wu W., Wang D., Lou W. (2019). Exosomal miRNA-106b from cancer-associated fibroblast promotes gemcitabine resistance in pancreatic cancer. Exp. Cell Res..

[48] Takikawa T., Masamune A., Yoshida N., Hamada S., Kogure T., Shimosegawa T. (2017). Exosomes Derived From Pancreatic Stellate Cells: MicroRNA Signature and Effects on Pancreatic Cancer Cells. Pancreas.

[49] Cao W., Zeng Z., He Z., Lei S. (2021). Hypoxic pancreatic stellate cell-derived exosomal mirnas promote proliferation and invasion of pancreatic cancer through the PTEN/AKT pathway. Aging.

[50] Ali S., Suresh R., Banerjee S., Bao B., Xu Z., Wilson J., Philip P.A., Apte M., Sarkar F.H. (2015). Contribution of microRNAs in understanding the pancreatic tumor microenvironment involving cancer associated stellate and fibroblast cells. Am. J. Cancer Res..

[51] Han S., Gonzalo D.H., Feely M., Rinaldi C., Belsare S., Zhai H., Kalra K., Gerber M.H., Forsmark C.E., Hughes S.J. (2018). Stroma-derived extracellular vesicles deliver tumor-suppressive miRNAs to pancreatic cancer cells. Oncotarget.

[52] Wang B., Xu Y., Wei Y., Lv L., Liu N., Lin R., Wang X., Shi B. (2021). Human Mesenchymal Stem Cell-Derived Exosomal microRNA-143 Promotes Apoptosis and Suppresses Cell Growth in Pancreatic Cancer via Target Gene Regulation. Front. Genet..

[53] Yao X., Mao Y., Wu D., Zhu Y., Lu J., Huang Y., Guo Y., Wang Z., Zhu S., Li X. (2021). Exosomal circ_0030167 derived from BM-MSCs inhibits the invasion, migration, proliferation and stemness of pancreatic cancer cells by sponging miR-338-5p and targeting the Wif1/Wnt8/beta-catenin axis. Cancer Lett..

[54] Shang S., Wang J., Chen S., Tian R., Zeng H., Wang L., Xia M., Zhu H., Zuo C. (2019). Exosomal miRNA-1231 derived from bone marrow mesenchymal stem cells inhibits the activity of pancreatic cancer. Cancer Med..

[55] Cione E., Cannataro R., Gallelli L., De Sarro G., Caroleo M.C. (2021). Exosome microRNAs in Metabolic Syndrome as Tools for the Early Monitoring of Diabetes and Possible Therapeutic Options. Pharmaceuticals.

[56] He X., Kuang G., Wu Y., Ou C. (2021). Emerging roles of exosomal miRNAs in diabetes mellitus. Clin. Transl. Med..

[57] Machida T., Tomofuji T., Maruyama T., Yoneda T., Ekuni D., Azuma T., Miyai H., Mizuno H., Kato H., Tsutsumi K. (2016). miR1246 and miR4644 in salivary exosome as potential biomarkers for pancreatobiliary tract cancer. Oncol. Rep..

[58] Xu Y.F., Hannafon B.N., Zhao Y.D., Postier R.G., Ding W.Q. (2017). Plasma exosome miR-196a and miR-1246 are potential indicators of localized pancreatic cancer. Oncotarget.

[59] Lai X., Wang M., McElyea S.D., Sherman S., House M., Korc M. (2017). A microRNA signature in circulating exosomes is superior to exosomal glypican-1 levels for diagnosing pancreatic cancer. Cancer Lett..

[60] Goto T., Fujiya M., Konishi H., Sasajima J., Fujibayashi S., Hayashi A., Utsumi T., Sato H., Iwama T., Ijiri M. (2018). An elevated expression of serum exosomal microRNA-191, -21, -451a of pancreatic neoplasm is considered to be efficient diagnostic marker. BMC Cancer.

[61] Wu L., Zhou W.B., Zhou J., Wei Y., Wang H.M., Liu X.D., Chen X.C., Wang W., Ye L., Yao L.C. (2020). Circulating exosomal microRNAs as novel potential detection biomarkers. Oncol. Lett..

[62] Chen J., Yao D., Chen W., Li Z., Guo Y., Zhu F., Hu X. (2022). Serum exosomal miR-451a acts as a candidate marke. Int. J. Biol. Markers.

[63] Kawamura S., Iinuma H., Wada K., Takahashi K., Minezaki S., Kainuma M., Shibuya M., Miura F., Sano K. (2019). Exosome-encapsulated microRNA-4525, microRNA-451a and microRNA-21 in portal vein blood is a high-sensitive liquid biomarker for the selection of high-risk pancreatic ductal adenocarcinoma patients. J. Hepatobiliary Pancreat. Sci..

[64] Nakamura S., Sadakari Y., Ohtsuka T., Okayama T., Nakashima Y., Gotoh Y., Saeki K., Mori Y., Nakata K., Miyasaka Y. (2019). Pancreatic Juice Exosomal MicroRNAs as Biomarkers for Detection of Pancreatic Ductal Adenocarcinoma. Ann. Surg. Oncol..

[65] Que R., Ding G., Chen J., Cao L. (2013). Analysis of serum exosomal microRNAs and clinicopathologic features of patients with pancreatic adenocarcinoma. World J. Surg. Oncol..

[66] Nesteruk K., Levink I.J.M., de Vries E., Visser I.J., Peppelenbosch M.P., Cahen D.L., Fuhler G.M., Bruno M.J. (2022). Extracellular vesicle-derived microRNAs in pancreatic juice as biomarkers for detection of pancreatic ductal adenocarcinoma. Pancreatology.

[67] Marin A.M., Mattar S.B., Amatuzzi R.F., Chammas R., Uno M., Zanette D.L., Aoki M.N. (2022). Plasma Exosome-Derived microRNAs as Potential Diagnostic and Prognostic Biomarkers in Braziliaatients. Biomolecules.

[68] Chen S.L., Ma M., Yan L., Xiong S.H., Liu Z., Li S., Liu T., Shang S., Zhang Y.Y., Zeng H. (2019). Clinical significance of exosomal miR-1231 in pancreatic cancer. Chin. J. Oncol..

[69] Lakhter A.J., Pratt R.E., Moore R.E., Doucette K.K., Maier B.F., DiMeglio L.A., Sims E.K. (2018). Beta cell extracellular vesicle miR-21-5p cargo is increased in response to inflammatory cytokines and serves as a biomarker of type 1 diabetes. Diabetologia.

[70] Xie Y., Hang Y., Wang Y., Sleightholm R., Prajapati D.R., Bader J., Yu A., Tang W., Jaramillo L., Li J. (2020). Stromal Modulation and Treatment of Metastatic Local Intraperitoneal Triple miRNA/siRNA Nanotherapy. ACS Nano.

[71] Gilles M.E., Hao L., Huang L., Rupaimoole R., Lopez-Casas P.P., Pulver E., Jeong J.C., Muthuswamy S.K., Hidalgo M., Bhatia S.N. (2018). Personalized RNA Medicine for Pancreatic Cancer. Clin. Cancer Res..

[72] Ding Y., Cao F., Sun H., Wang Y., Liu S., Wu Y., Cui Q., Mei W., Li F. (2019). Exosomes derived from human umbilical cord mesenchymal stromal cells deliver exogenous miR-145-5p to inhibit pancreatic ductal adenocarcinoma progression. Cancer Lett..

[73] Zuo L., Tao H., Xu H., Li C., Qiao G., Guo M., Cao S., Liu M., Lin X. (2020). Exosomes-Coated miR-34a Displays Potent Antitumor Activity in Both in vitro and in vivo. Drug Des. Devel. Ther..

[74] Kamerkar S., LeBleu V.S., Sugimoto H., Yang S., Ruivo C.F., Melo S.A., Lee J.J., Kalluri R. (2017). Exosomes facilitate therapeutic targeting of oncogenic KRAS in pancreatic cancer. Nature.

[75] Su M.J., Aldawsari H., Amiji M. (2016). Cell Exosome-Mediated Macrophage Reprogramming and the Role of MicroRNAs 155 and 125b2 Transfection using Nanoparticle Delivery Systems. Sci. Rep..

